# Diagnostic performance of ^68^Ga-PSMA-11 PET/MRI-guided biopsy in patients with suspected prostate cancer: a prospective single-center study

**DOI:** 10.1007/s00259-021-05261-y

**Published:** 2021-02-23

**Authors:** Daniela A. Ferraro, Anton S. Becker, Benedikt Kranzbühler, Iliana Mebert, Anka Baltensperger, Konstantinos G. Zeimpekis, Hannes Grünig, Michael Messerli, Niels J. Rupp, Jan H. Rueschoff, Ashkan Mortezavi, Olivio F. Donati, Marcelo T. Sapienza, Daniel Eberli, Irene A. Burger

**Affiliations:** 1 Department of Nuclear Medicine, University Hospital Zurich, University of Zurich, Zurich, Switzerland; 2 Department of Radiology and Oncology, Faculdade de Medicina FMUSP, Universidade de Sao Paulo, Sao Paulo, Brazil; 3 Institute of Interventional and Diagnostic Radiology, University Hospital Zurich, University of Zurich, Zurich, Switzerland; 4 Department of Radiology, Memorial Sloan Kettering Cancer Center, New York City, NY USA; 5 Department of Urology, University Hospital Zürich, University of Zurich, Zurich, Switzerland; 6 Department of Pathology and Molecular Pathology, University Hospital Zurich, University of Zurich, Zurich, Switzerland; 7 Department of Nuclear Medicine, Kantonsspital Baden, Baden, Switzerland; 8 Department of Nuclear Medicine, University Hospital Zürich, Rämistrasse 100, 8091 Zürich, Switzerland

**Keywords:** Imaging-guided biopsy, PET/MR, Prostate biopsy, PSMA-PET accuracy, Targeted biopsy, Template biopsy

## Abstract

**Purpose:**

Ultrasound-guided biopsy (US biopsy) with 10–12 cores has a suboptimal sensitivity for clinically significant prostate cancer (sigPCa). If US biopsy is negative, magnetic resonance imaging (MRI)–guided biopsy is recommended, despite a low specificity for lesions with score 3–5 on Prostate Imaging Reporting and Data System (PIRADS). Screening and biopsy guidance using an imaging modality with high accuracy could reduce the number of unnecessary biopsies, reducing side effects. The aim of this study was to assess the performance of positron emission tomography/MRI with ^68^Ga-labeled prostate-specific membrane antigen (PSMA-PET/MRI) to detect and localize primary sigPCa (ISUP grade group 3 and/or cancer core length ≥ 6 mm) and guide biopsy.

**Methods:**

Prospective, open-label, single-center, non-randomized, diagnostic accuracy study including patients with suspected PCa by elevation of prostate-specific antigen (PSA) level and a suspicious lesion (PIRADS ≥3) on multiparametric MRI (mpMRI). Forty-two patients underwent PSMA-PET/MRI followed by both PSMA-PET/MRI-guided and section-based saturation template biopsy between May 2017 and February 2019. Primary outcome was the accuracy of PSMA-PET/MRI for biopsy guidance using section-based saturation template biopsy as the reference standard.

**Results:**

SigPCa was found in 62% of the patients. Patient-based sensitivity, specificity, negative and positive predictive value, and accuracy for sigPCa were 96%, 81%, 93%, 89%, and 90%, respectively. One patient had PSMA-negative sigPCa. Eight of nine false-positive lesions corresponded to cancer on prostatectomy and one in six false-negative lesions was negative on prostatectomy.

**Conclusion:**

PSMA-PET/MRI has a high accuracy for detecting sigPCa and is a promising tool to select patients with suspicion of PCa for biopsy.

**Trial registration:**

This trial was retrospectively registered under the name “Positron Emission Tomography/Magnetic Resonance Imaging (PET/MRI) Guided Biopsy in Men with Elevated PSA” (NCT03187990) on 06/15/2017 (https://clinicaltrials.gov/ct2/show/NCT03187990).

**Supplementary Information:**

The online version contains supplementary material available at 10.1007/s00259-021-05261-y.

## Introduction

Assessment of histological tumor grade on biopsy is needed for diagnosis and risk classification of prostate cancer (PCa). The updated European Association of Urology (EAU) guideline recommends ultrasound-guided systematic prostate biopsy (US biopsy) in patients with suspicion of PCa [1, 2]. Magnetic resonance imaging (MRI)–guided biopsy is considered for cases in which no cancer was detected [2]. The PROMIS trial revealed sensitivity of only 48% for their primary definition of clinically significant cancer (sigPCa) using 10–12 cores US biopsy and suggested that, instead, multiparametric MRI (mpMRI) should be used to reduce the number of unnecessary biopsies. However, if all lesions with a score ≥ 3 on Prostate Imaging Reporting and Data System (PIRADS) are targeted, the specificity of mpMRI is only 41% [3]. Several other studies also showed superior detection rates of sigPCa in MRI-guided biopsy compared to US biopsy [4–7]. Nevertheless, false-negative results or histological upgrade after surgery are found in 21% of patients [8–10]. The most reliable method to reduce undersampling and false-negative results is transperineal saturation biopsy (template biopsy) with samples taken from all 20 Barzell zones, leading to organ coverage of approximately 95% [10]. Screening and imaging-guided biopsy could potentially reduce side effects of saturation prostate biopsies [11], but recent studies suggest that a template-based systematic approach should not be omitted despite mpMRI [6, 12].

Positron emission tomography (PET)/MRI targeting prostate-specific membrane antigen (PSMA) could be an ideal technique to improve the accuracy of imaging-guided biopsies, combining the high sensitivity and specificity of PSMA-PET for PCa with the high anatomical contrast and spatial resolution of MRI [13–15]. Despite promising results in PSMA-PET/computed tomography (CT) for biopsy targeting [16], with an accuracy of 80.6% for sigPCa [17], the diagnostic accuracy of PSMA-PET/MRI-guided biopsy has not yet been prospectively assessed. Therefore, the aim of this study is to assess the performance of ^68^Ga-PSMA-11 PET/MRI (PSMA-PET/MRI) to detect and localize primary sigPCa for accurate prostate biopsy guidance.

## Patients and methods

### Study design

**Fig. 1:**
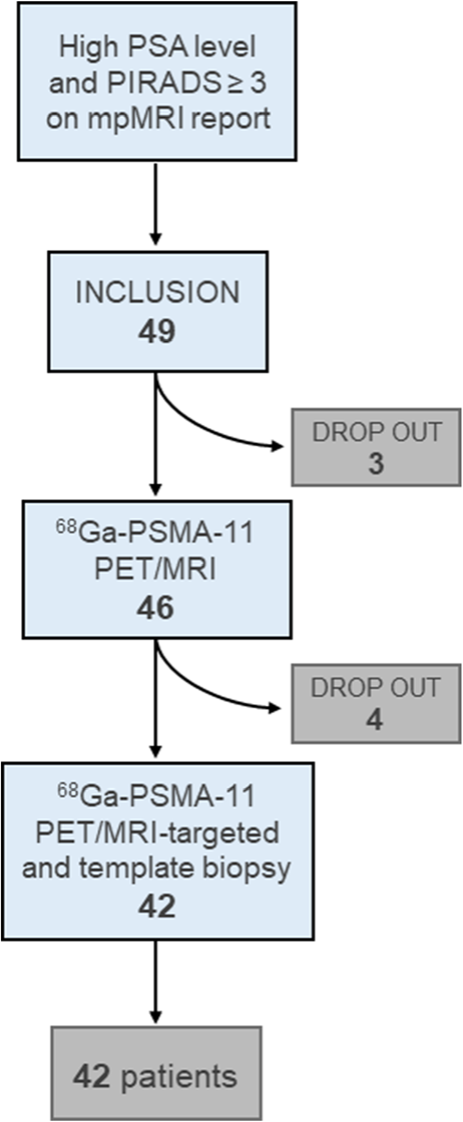
Patient selection and inclusion in the study

The study was designed as an open-label, single-center, non-randomized, prospective diagnostic accuracy study including patients with suspected PCa. Patients without biopsy-proven sigPCa but suspicion of cancer due to persistently elevated prostate-specific antigen (PSA) (PSA > 2.5 ng/ml if age 30–50 years and PSA > 4 ng/ml if age 50–80 years) and at least one suspicious lesion on mpMRI clinical report (PIRADS ≥3) were included. All patients underwent PSMA-PET/MRI followed by both PSMA-PET/MRI-guided and section-based saturation template biopsy of the prostate between May 2017 and February 2019. Exclusion criteria were age < 30 and > 80, previous biopsy within 8 weeks prior to imaging, previous pelvic irradiation, prostatectomy, transurethral resection of the prostate (TURP) or androgen deprivation hormonal therapy (ADT), and any contra-indication to MRI or prostate biopsy as well active urinary tract infection or indwelling catheter. PSMA-PET/MRI and biopsy were performed with an interval of up to 30 weeks from mpMRI (median 2.7 weeks, IQR 0.4–12). Figure 1 illustrates patient selection. This study was approved by the institutional review board (BASEC Nr: 2017-00016), was carried out in accordance with the Declaration of Helsinki, and is registered in the international trial registry ClinicalTrials.gov (NCT03187990).

### ^68^Ga-PSMA-11 PET/MRI imaging acquisition and analysis

All patients underwent a pelvic PET/MRI on a hybrid scanner (SIGNA PET/MR, GE Healthcare, Waukesha, WI, USA) 60 min after injection of 85 MBq of ^68^Ga-PSMA-11. A 15-min frame over the prostate was recorded, allowing reducing the dose since patients without confirmed cancer were included. For biopsy targeting, suspected lesions were delineated on PSMA-PET/MRI by a double-board-certified nuclear medicine physician and radiologist, specialist in pelvic imaging, with 10 and 5 years of experience (IAB,MM), with a maximum of three target lesions. Imaging protocol and analysis are given in the supplements (Online Resource 1).

### Biopsy

Biopsies were performed under general anesthesia by specialized urologists with US-MRI software fusion (BiopSee®). Axial fused PSMA-PET/MRI images in DICOM format were uploaded to BiopSee® instead of T2w MRI sequences. Standard transperineal template biopsy with number of cores adapted to prostate volume as well as PSMA-PET/MRI-targeted biopsy was performed with a maximum of three cores per target lesion (Online Resource 2). Patients with no suspicious uptake on PSMA-PET/MRI or with discordant lesions between PSMA-PET/MRI and mpMRI underwent template biopsy and the urologist was free to target any suspicious lesion on mpMRI.

### Clinically significant cancer definition

SigPCa was defined as International Society of Urological Pathology (ISUP) grade group 3 and/or cancer core length ≥ 6 mm [18]. Conversely, clinically insignificant cancer (insigPCa) was defined as ISUP 1 or 2 lesions with cancer core length < 6 mm. Biopsies with the latter characteristics were classified as negative for further analysis. Results based on other definition of sigPCa (ISUP ≥2) are in Table S3 (Online Resource 1).

### Reference standard

Results of PSMA-PET/MRI-targeted biopsies were compared to template biopsies regarding presence of sigPCa on histopathology. All patients classified as having a false-positive or false-negative ^68^Ga-PSMA-11 PET/MRI result had the biopsy samples, or radical prostatectomy (RPE) specimens if available, reevaluated on histopathology for possible explanations including PSMA immunohistochemistry (IHC). Biopsies and RPE specimens were evaluated by two board-certified genitourinary pathologists (NR, JR) with 8–10 years of experience.

### Data analysis

Study results were analyzed using descriptive statistics and frequency tables in Excel (Excel2016, Microsoft, USA). Accuracy was assessed on 2 × 2 contingency tables on patient and lesion basis. For lesion-based analysis, the number of lesions was defined as number of PSMA-positive lesions added to number of PSMA-negative lesions with sigPCa found on biopsy. For patient-based analysis in patients with more than one lesion and different classifications (for example, one true-positive and one false-negative lesion), we considered whether PSMA-PET/MRI correctly staged the patient regarding the presence or absence of sigPCa according to Table S1 (Online Resource 1). We also assessed patient-based accuracy for PET/MRI-targeted cores.

## Results

### General

Forty-nine patients met the inclusion criteria and were included between May 2017 and January 2020. Seven patients withdrew participation before the PSMA-PET/MRI scan or the biopsy was performed; therefore, data from 42 patients were analyzed (descriptive characteristics in Table 1). Median interval between PSMA-PET/MRI and biopsy was 12 days (interquartile range (IQR) 6–18).

**Table 1:** Characteristics of the patients at inclusion in the study (*n* = 42)

Characteristics	Value
Age at scan (years)	
Mean ± SD	64 ± 6
Median (IQR)	65 (59–68)
PSA at time of PET scan (ng/ml)	
Mean ± SD	10 ± 7.4
Median (IQR)	8 (7–11)
PIRADS (*n*)	
3	7 (16.7%)
4	24 (57.1%)
5	11 (26.2%)

SD = standard deviation; IQR = interquartile range

### Biopsy

**Fig. 2:**
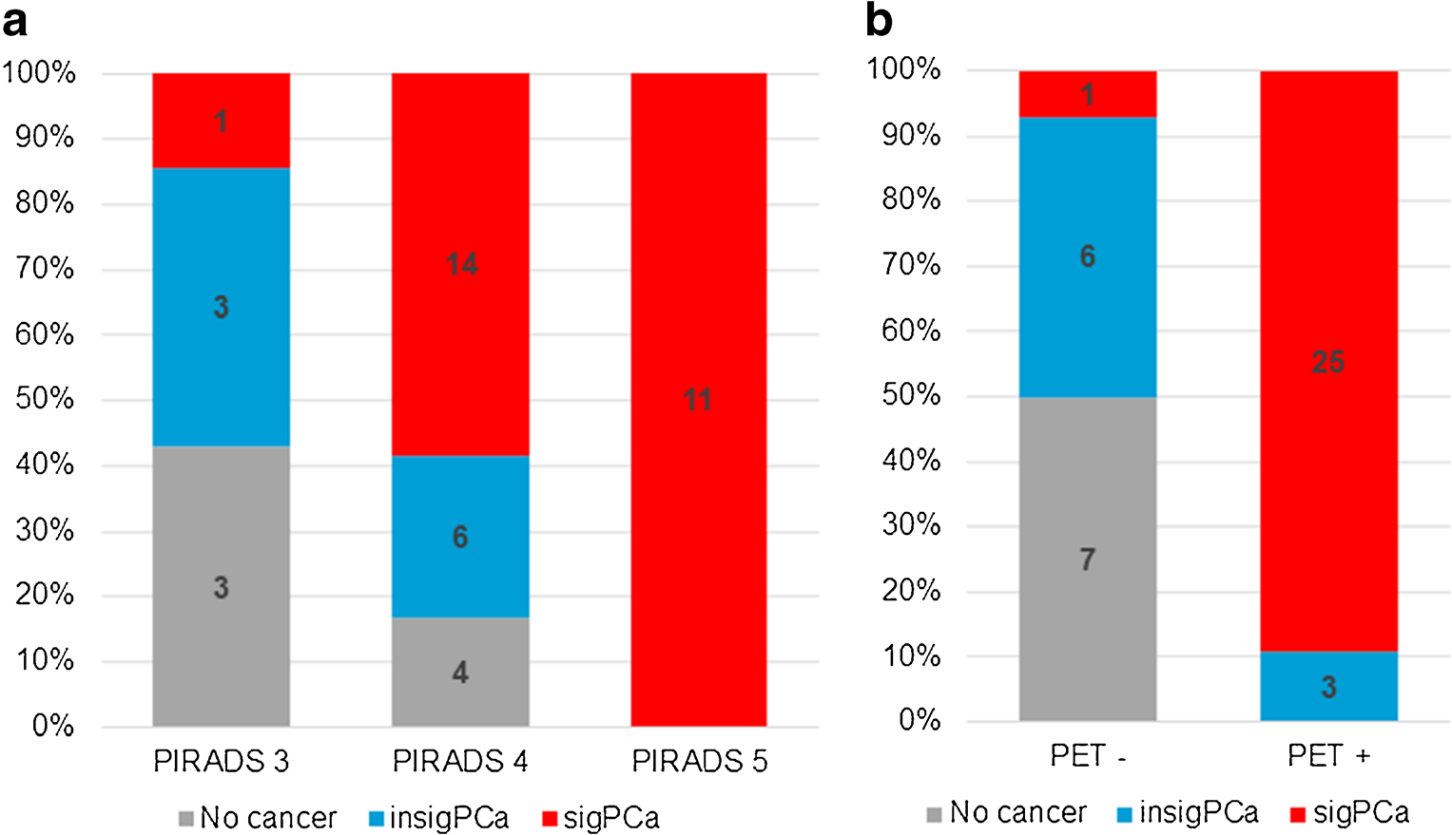
Distribution of patients with clinically significant prostate cancer (sigPCa), clinically insignificant prostate cancer (insigPCa), and no evidence of disease on biopsy in correlation to PIRADS classification on multiparametric resonance magnetic imaging (**a**) and ^68^Ga-PSMA-11 PET/MRI result (**b**)

Based on template and targeted biopsy, 26 of 42 (62%) patients had sigPCa. While there was no malignancy in seven of 42 patients (17%), in the remaining nine patients (21.4%), cancer detected on biopsy did not meet the criteria of sigPCa. Fifteen cases of sigPCa were detected by both template and targeted biopsies (58%, 15/26), nine only by template (35%) and two only by targeted (8%). Two cases of insigPca were detected by both biopsy methods (22%, 2/9), six only by template (67%) and one only by targeted. Table 2 and Fig. 2 show the distribution of sigPCa, insigPCa, and no disease, in correlation to PIRADS, ISUP, and PSMA-PET/MRI result. Eighteen patients had one lesion, seven patients had two, and one patient had three lesions, resulting in 35 sigPCa lesions in total. The median number of positive cores per patient was three (IQR 2–6). The median number of samples taken per patient was 43 (IQR 36–44). Eight patients (19%, 8/42) had biopsy procedure complications, none life-threatening. Six patients presented to the emergency department for acute urinary retention, one patient had postinterventional bleeding with need of catheter irrigation, and one patient with anesthesia complications was admitted for observation and released the day after.

**Table 2:** Distribution of patients with sigPCa and insigPCa, based on biopsy, according to ISUP grade groups. Clinically significant prostate cancer defined as ISUP grade ≥ 3 and/or cancer core length ≥ 6 mm. Seven patients had no cancer on biopsy

	sigPCa	insigPCa
ISUP		
1	1 (4%)	3 (33%)
2	6 (23%)	6 (67%)
3	9 (34%)	–
4	8 (31%)	–
5	2 (8%)	–
Total	26	9

sigPCa = clinically significant prostate cancer; insigPCa = clinically insignificant prostate cancer

### ^68^Ga-PSMA-11 PET/MRI

**Fig. 3:**
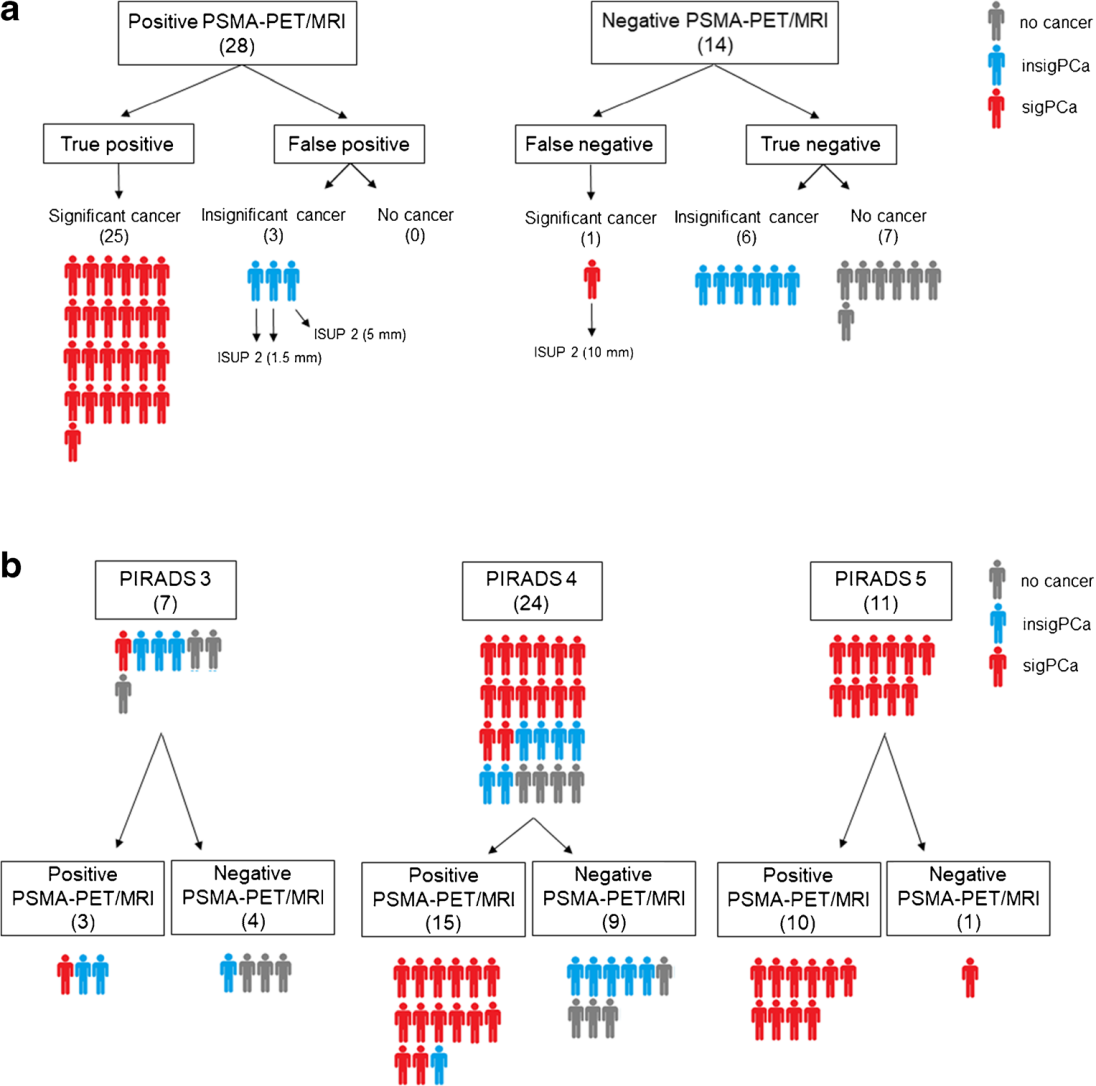
Distribution of patients with clinically significant prostate cancer (sigPCa), clinically insignificant prostate cancer (insigPCa), and no evidence of disease on biopsy according to ^68^Ga-PSMA-11 PET/MRI results (**a**) and according to ^68^Ga-PSMA-11 PET/MRI results in correlation to PIRADS classification on multiparametric resonance magnetic imaging (**b**). The single false-negative case and the three false-positive cases shown in part “**a**” are shown in part “**b**” under PIRADS 5/negative PSMA-PET/MRI and PIRADS 3/positive PSMA-PET/MRI (two cases) and 4/positive PSMA-PET/MRI (one case), respectively

Table 3 shows sensitivity, specificity, positive predictive value (PPV), negative predictive value (NPV), and accuracy of PSMA-PET/MRI per patient and per lesion. PSMA-PET/MRI was positive in 28 patients (66.7%, 28/42), of which 25 had sigPCa on biopsy (89%, 25/28) and negative in 14 patients (33.3%, 14/42), of which only one had sigPCa (7%, 1/14) (Figs. 2b and 3a). Nineteen patients had one PSMA-positive lesion, eight patients had two lesions, and one patient had three lesions, resulting in 38 PSMA-positive lesions. One patient had a lesion without PSMA uptake but clear PIRADS 5 features on MRI, confirmed as sigPCa by MRI-targeted biopsy and classified as negative PSMA-PET/MRI for further analysis. Figure 3b shows PSMA-PET/MRI results in relation to PIRADS.

**Table 3:** Performance of PSMA-PET/MRI for biopsy guidance, given patient-based for PSMA-PET/MRI imaging findings and PET-targeted cores, and lesion-based

	Patient-based	Patient-based targeted cores	Lesion-based
Sensitivity	96% (25/26)	65% (17/26)	83% (29/35)
Specificity	81% (13/16)	81% (13/16)	–
PPV	89% (25/28)	61% (17/28)	76% (29/38)
NPV	93% (13/14)	93% (13/14)	–
Accuracy	90% (38/42)	71% (30/42)	–

PPV = positive predictive value; NPV = negative predictive value. For the targeted core analysis, values were calculated as if patients with a negative PSMA-PET/MRI were not submitted to biopsy and patients with a positive PSMA-PET/MRI underwent only PSMA-PET/MRI-targeted biopsy. Lesion-based specificity and NPV were not calculated since patients with negative PSMA-PET/MRI and no significant cancer on biopsy have, per definition, no lesion

The accuracy of PSMA-targeted cores was lower compared to PSMA-PET/MRI imaging findings. In eight cases with PSMA uptake in the sigPCa lesion, the three target needles were negative, but additional adjacent template needles confirmed sigPCa.

Per lesion, 44 lesions were detected in 29 patients (38 on PSMA-PET/MRI and 35 on biopsy, with 29 concordant lesions). Six sigPCa lesions and 24 insigPCa lesions were not detected by PSMA-PET/MRI.

### False-positive PSMA-PET/MRI

**Fig. 4:**
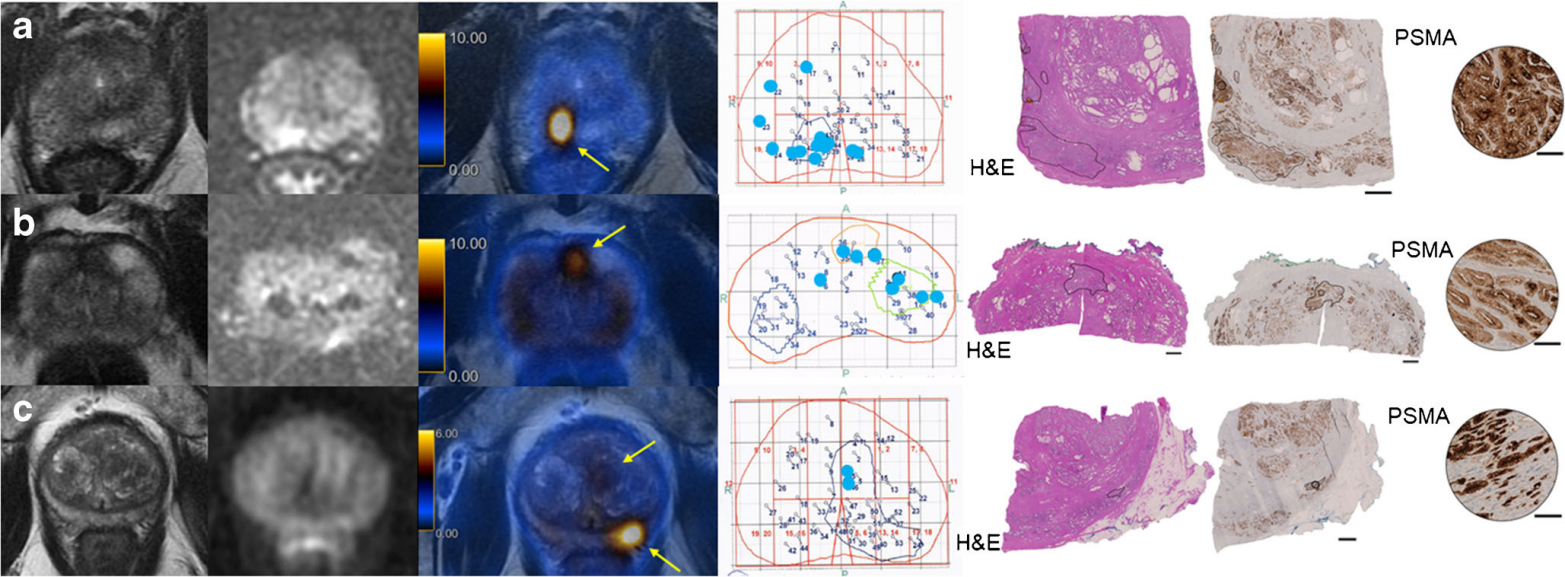
All three patients with a false-positive PSMA-PET/MRI. From left to right, prostate MRI sequences T2-weighted and diffusion-weighted images (*b* value 1000), fused PET/MRI, representative pathology map with biopsy results, and radical prostatectomy (RPE) specimen with tumor outlined on hematoxylin and eosin staining (H&E) and PSMA-IHC (overview and magnification). Bars represent 2.5 mm in the H&E and PSMA-IHC images and 100 μm in the PSMA-IHC magnified images. Blue dots in the pathology map correspond to location of needles with clinically insignificant cancer diagnosed. **a** 67-year-old patient, with a PSA of 7.3 ng/ml and a PIRADS 4 lesion on mpMRI. PSMA-PET/MRI shows one targeted lesion (arrow) in the posterior right peripheral zone, where biopsy found ISUP grade group 2 tumor with up to 1.5-mm length. RPE specimen shows a PSMA-positive ISUP grade group 3 tumor in the PSMA uptake area. **b** 65-year-old patient, with a PSA of 7.18 ng/ml and a PIRADS 3 lesion on mpMRI. PSMA-PET/MRI shows one targeted lesion (arrow) in the anterior zone, where biopsy found ISUP grade group 2 tumor with up to 1.5-mm length. RPE specimen shows a PSMA-positive ISUP grade group 2 tumor in the PSMA uptake area. **c** 65-year-old patient, with a PSA of 48.5 ng/ml and a PIRADS 3 lesion on mpMRI. PSMA-PET/MRI shows two targeted lesions (arrows) in the transition zone corresponding on biopsy to ISUP grade group 2 tumor up to 5 mm length, and in the posterior left peripheral zone, where biopsy was negative. RPE specimen shows a PSMA-positive ISUP grade group 3 tumor in the PSMA uptake area of the posterior left peripheral zone

Three patients had a false-positive PSMA-PET/MRI, but insigPCa on biopsy in at least one of the PSMA uptake areas (ISUP grade group 2 with cancer length of 1.5–5 mm). Relevant cancer was confirmed on RPE specimen in all three cases (Fig. 4).

Per lesion, nine lesions were false-positive (Online Resource 3). In all patients, RPE was available and showed cancer in eight lesions (Table 4). In the case without cancer, additional pathology workup showed clear PSMA overexpression on IHC, but no benign or malignant alterations. Interval between biopsy and RPE in these patients ranged from 1 to 3.8 months.

**Table 4:** Findings on PET (SUV_max_), biopsy, and radical prostatectomy (RPE) specimen of the false-positive and false-negative lesions on PSMA-PET/MRI. PSMA-PET/MRI image of each lesion can be seen in the correspondent supplementary figure (Online Resources 3 for Fig. S2 and 4 for Fig. S3) showed in the first column

	Fig.	SUV_max_	Biopsy			RPE specimen	
			Finding	ISUP	Length (mm)	Finding	ISUP
False-positive lesions*							
Pat. 03	S2 a	7.9	ASAP	–	–	PSMA overexpression	–
Pat. 24	S2 b	5.3	Inflammation	–	–	Cancer	3
Pat. 30	S2 c	5.4	insigPCa	2	1.0	Cancer	2
Pat. 32	S2 d	12.9	insigPCa	2	2.0	Cancer	2
Pat. 33	S2 e	9.4	insigPCa	2	1.5	Cancer	3
Pat. 35	S2 f	4.4	insigPCa	2	5.0	Cancer	2
Pat. 35	S2 g	5.7	None	–	–	Cancer	3
Pat. 38	S2 h	10.1	None	–	–	Cancer	2
Pat. 42	S2 i	8	insigPCa	2	1.5	Cancer	2
False-negative lesions*							
Pat. 05	S3 a	–	sigPCa	1	6.0	Not available	–
Pat. 07	S3 b	–	sigPCa	3	1.0	No cancer	–
Pat. 16	S3 c	–	sigPCa	3	3.0	Cancer	3
Pat. 26	S3 d	–	sigPCa	4	6.0	Cancer	2
Pat. 32	S3 e	–	sigPCa	2	6.0	Cancer	2
Pat. 39	S3 f	–	sigPCa PSMA-negative	2	10.0	Not available	–

*Based on biopsy findings

ASAP = atypical small acinar proliferation; insigPCa = clinically insignificant prostate cancer; sigPCa = clinically significant prostate cancer; SUVmax = maximum standardized uptake value

### False-negative PSMA-PET/MRI

**Fig. 5:**
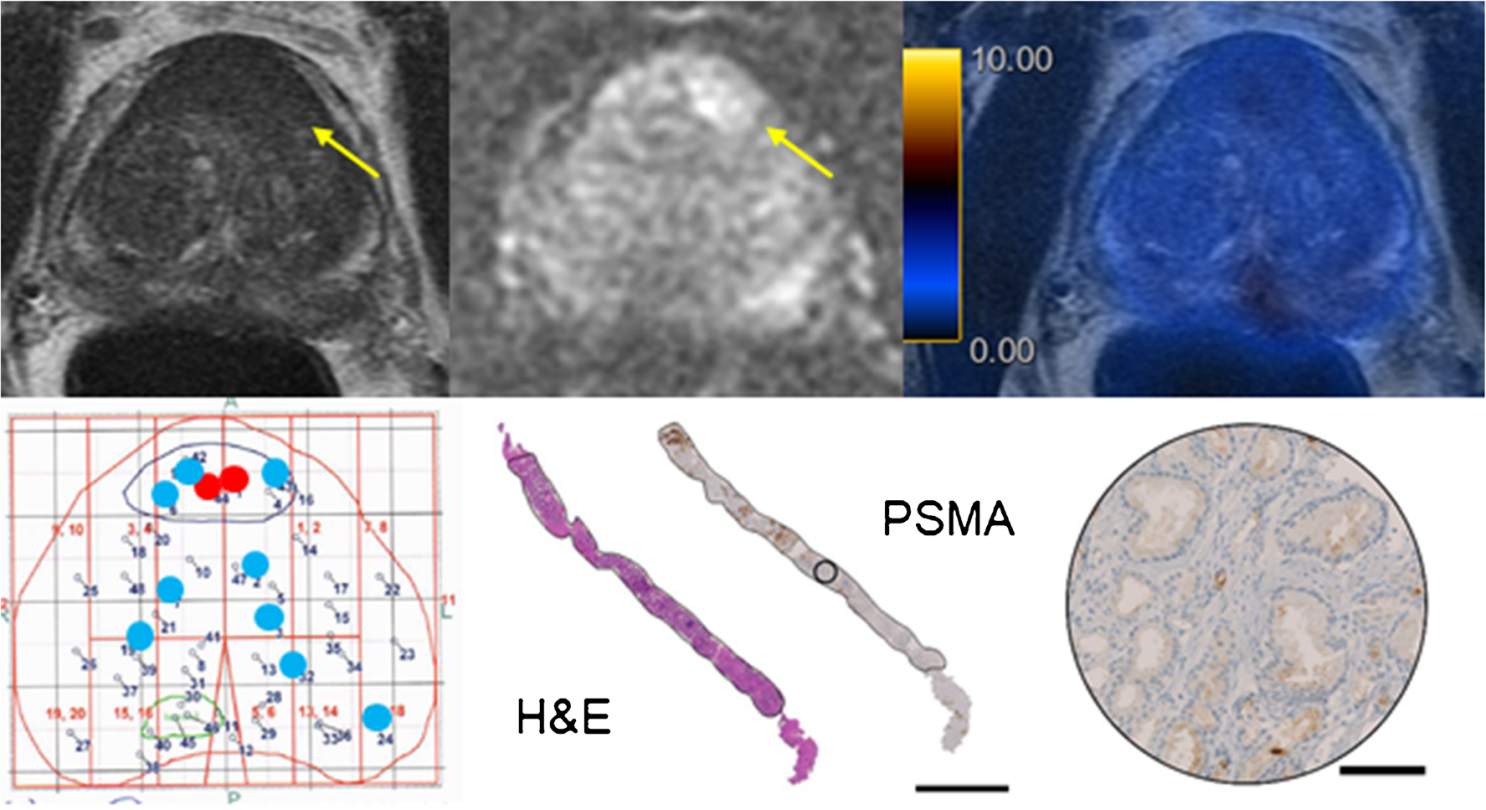
The only patient with a false-negative PSMA-PET/MRI in our cohort. A 62-year-old patient with a PSA of 11.38 ng/ml. Top images from left to right are prostate MRI sequences T2-weighted and diffusion-weighted images and fused PET/MRI showing a PIRADS 5 lesion in the anterior transition zone (arrows) with no PSMA uptake. Bottom left image shows the representative pathology map with biopsy results including two cores with clinically significant cancer in the lesion area (red dots, ISUP grade group 2 tumor with length up to 10 mm) and many cores with clinically insignificant cancer (blue dots). Remaining bottom images show one of the biopsy cores with clinically significant cancer. The tumor is outlined in hematoxylin and eosin staining (H&E) and PSMA-IHC (overview and magnification), showing a virtually PSMA-negative tumor. Bars represent 2.5 mm in the H&E and PSMA-IHC images and 100 μm in the PSMA-IHC magnified image

^68^Ga-PSMA-11 PET/MRI was false-negative in one patient with sigPCa, who had two positive cores with ISUP grade group 2 and lengths of 9 and 10 mm. Despite no PSMA uptake, the lesion was easily appreciated on T2- and diffusion-weighted sequences of the MRI component (Fig. 5).

Per lesion, six were false-negatives (Online Resource 4). In four lesions, ISUP grade was low or tumor volume small (up to 3 mm) on biopsy. In one case, there was no cancer on RPE in the corresponding location of the positive biopsy core (Table 4). One lesion with positive cores of ISUP grade group 4 (6 mm) was downgraded to ISUP grade 2 on RPE and in one lesion (ISUP 2, 10 mm) biopsy cores stained for PSMA on IHC showed a PSMA-negative tumor (Fig. 5). The interval between biopsy and RPE in these patients ranged from 1.4 to 3.7 months.

## Discussion

PSMA-PET/MRI showed a patient-based accuracy of 90% for detecting sigPCa in our cohort, with a sensitivity of 96% and specificity of 81%. This is higher than the mpMRI accuracy reported in most studies using template biopsy as reference standard [19], including the PROMIS trial, which reported sensitivity and specificity of 93% and 41%, respectively [3]. Our improved specificity was mainly due to PSMA-PET mitigating false-positive mpMRI PIRADS 3 and 4 lesions harboring no sigPCa (Fig. 3b). The PROMIS authors conclude that screening by mpMRI prior to biopsy could reduce the number of unnecessary biopsies. Our study suggests PSMA-PET/MRI could further improve on mpMRI patient selection.

In our cohort, 16 patients (38%) without sigPCa underwent biopsy based on equivocal or suspicious lesions on mpMRI (PIRADS 3 to 5). Omitting biopsy in patients with negative PSMA-PET/MRI would have spared 13 (13/16, 81%), without missing any patient with sigPCa. Only one patient had a false-negative PSMA-PET result; however, his ISUP 2 tumor would not have been missed due to clear PIRADS 5 features on MRI. The tumor was PSMA-negative on IHC, which is in accordance with the reported rate of around 5% of PSMA-negative tumors in the literature [20]. For the three patients with false-positive PSMA-PET/MR results, insigPCa was present on template biopsy, and cancer with Gleason 4 pattern was confirmed on RPE in each case.

Interestingly, despite PET findings confirmed by biopsy in 90% of the cases, the accuracy of 71% with a sensitivity of 65% for PET-targeted biopsy shows that some of the sigPCa lesions seen on PET are actually missed by the three targeted cores. This was already reported by van der Leest et al. [9] in a study comparing transrectal US-guided biopsy versus MRI-guided biopsy. They found that positive TRUS cores were obtained from the mpMRI lesion area or its neighboring and suggested that four additional perilesional cores greatly improved sigPCa detection with MRI-guided biopsy. They concluded that the majority of sigPCa missed by targeted biopsy was probably due to sampling errors related to spatial heterogeneity of the tumor [9].

False-negative and false-positive lesions in our study were often lesions with borderline characteristics regarding clinical significance. The lower PSMA expression in Gleason pattern 3 compared to 4 has been demonstrated on IHC [20–22] and our results probably reflect it: most false-negative lesions corresponded to low-grade groups (ISUP 1 and 2) or small volume tumors and, in only one case, a significant PSMA-negative tumor. Omitting template biopsy in our cohort would leave undetected six sigPCa, but also 24 lesions with insigPCa, mitigating overdiagnosis. On the other hand, eight of nine false-positive lesions based on biopsy were insigPCa, with only one showing no cancer on RPE specimen. This case was previously published as a case report with extensive histopathology workup excluding inflammation, pre-cancerous lesions, or other malignancies [23]. Therefore, template and targeted biopsies were false-negative for significant disease for eight lesions.

The definition of sigPCa is not standardized among centers; therefore, we chose the definition used in the PROMIS trial [3] to allow a direct comparison of our results. We recognize that other definitions can be found in the literature and that more recent guidelines of the EAU suggest considering any ISUP grade group 2 biopsy as sigPCa [1, 2]. Incorporating this cutoff, we would have had only one false-positive PET in our cohort on per-patient analysis, but four false-negative PET scans. Therefore, we also give the results using this other definition of sigPCa in Tables S2 and S3 (Online Resource 1).

There is sparse literature on PSMA-PET/CT-guided biopsy. Recently, PSMA-PET/CT was compared, for biopsy purposes, to micro-ultrasound, a novel imaging technique with promising results when added to mpMRI [24]. PSMA-PET/CT yielded an accuracy of 83% versus 61% of micro-ultrasound [25]. No study so far compared PSMA-PET/CT to PET/MRI for biopsy guidance. In our limited experience (anecdotal data not included in present study), delineating the prostate and PSMA-positive lesions on non-contrast-enhanced CT using US-fusion-software is feasible but cumbersome. In a study with 31 patients, sensitivity and specificity for sigPCa of PSMA-PET/CT-guided biopsy was 100% and 68% [17]. The higher sensitivity and lower specificity compared to our study may be explained by the approach to biopsy the prostate area with highest PSMA uptake if no suspicious lesion was reported. This probably led to false-positives, which could be ruled out by MRI but not by CT, such as activity in the central zone [26]. Another study found a region-based sensitivity of PET/CT for sigPCa of 61%, lower than our lesion-based sensitivity (83%). However, patients did not undergo mpMRI so no insights on PET/CT limitations compared to PET/MRI could be drawn [27]. A prospective study showed higher detection rate of sigPCa for PET/CT compared to 12-core TRUS biopsy; however, biopsies were performed within the CT scanner, and again mpMRI was not performed routinely [28]. In a study with 97 patients that compared PSMA-PET/CT with mpMRI, PSMA-PET/CT identified 25% of patients with Gleason 7 tumors missed by mpMRI [29]. Due to their inclusion criteria, around half of the patients that were biopsied had contra-indications to mpMRI or PIRADS 1 or 2; what makes it difficult to compare their results to ours but rather offers nice complementary data. Interestingly, these results are similar to the results found by the same group in a smaller cohort using 11C-Choline PET/CT, with 26% of patients with Gleason 7 tumors detected by PET in patients with negative or contra-indication to mpMRI [30]. Advantages of PET/MRI over PET/CT are that surgeons are already used to delineate prostate and target lesions based on MRI and that they can target lesions by both PSMA-PET and MRI in case of discordance. That a combination of these both methods could further improve the sensitivity for detecting PCa was already shown by Eiber et al. [13]. While PET/MRI profits from the higher soft tissue contrast, studies performing head-to-head comparisons are needed to investigate whether this offsets the higher general availability and lower cost of PET/CT. Moreover, post hoc image fusion of MRI and PSMA-PET (from PET/CT) may be a viable option for centers without a dedicated PET/MRI device.

Despite the good coverage of template biopsy, absence of RPE specimen as reference standard in some cases is a limitation of this study. Given that RPE specimen were not available for all patients, we opted to use RPE results only to investigate false-positive or false-negative lesions on PSMA-PET/MRI. Another limitation is pre-selection of patients based on mpMRI results. The aim of this proof-of-mechanism study was to assess whether PSMA-PET/MR could add value to mpMRI. Given that the probability of sigPCa in patients with PIRADS 1–2 is very low, we opted to exclude those patients in a first step. However, this limits the conclusion about the accuracy of PET scans in an mpMRI naïve population.

## Conclusions

PSMA-PET/MRI has a high accuracy for detecting sigPCa and is a promising tool to select patients for biopsy as well as to guide it, with the potential to substantially reduce unnecessary biopsies compared to mpMRI and might even improve the detection of sigPCa in comparison to systematic template biopsies.

## Supplementary Information

ESM 1 (PDF 112 kb)

ESM 2 (PDF 275 kb)

ESM 3 (PDF 235 kb)

ESM 4 (PDF 235 kb)
