# Supplementary material for: Diagnostic performance of 68Ga-PSMA-11 PET/MRI-guided biopsy in patients with suspected prostate cancer: a prospective single-center study
Source: Eur J Nucl Med Mol Imaging. 2021 Feb 23;48(10):3315–24. doi: 10.1007/s00259-021-05261-y (PMC8426229; doi:10.1007/s00259-021-05261-y)
Supplement: Supplementary file 1 — (PDF 112 kb) [file 259_2021_5261_MOESM1_ESM.pdf]

Supplementary Material

Diagnostic performance of ^{68}Ga-PSMA-11 PET/MRI-guided biopsy in patients with suspected prostate cancer: a prospective single-center study

Journal: EJNMMI

Authors: *Daniela A. Ferraro, Anton S. Becker, Benedikt Kranzbühler, Iliana Mebert, Anka Baltensperger, Konstantinos G. Zeimpekis, Hannes Grünig, Michael Messerli, Niels J. Rupp, Jan H. Rueschoff, Ashkan Mortezavi, Olivio F. Donati, Marcelo T. Sapienza, Daniel Eberli, Irene A. Burger**

*Corresponding author:

Irene A. Burger

Department of Nuclear Medicine, University Hospital Zurich, University of Zurich, Zurich, Switzerland and Department of Nuclear Medicine, Kantonsspital Baden, Baden, Switzerland.

E-mail: irene.burger@usz.ch

Sample size calculation

The sample size was calculated with the assumption that the sensitivity of PSMA PET would be 15% higher than mpMRI. We determine the number of patients required to achieve a power of 80% to prove an increase of the sensitivity by 15% on a significance level of α=5% in this paired design by utilizing the one-sided MC Nemar test. We used the statistical programming language R to perform the sample size calculation and the result is that 40 patients are needed.

Imaging protocol

All patients underwent a pelvic PET/MRI on a dedicated hybrid scanner (SIGNA PET/MR, GE Healthcare, Waukesha, WI, USA) 60 minutes after injection of 85 MBq PSMA and images were transferred to a dedicated review workstation (Advantage Workstation, Version 4·6 or 4·7, GE Healthcare) for reporting. To reduce PSMA activity in the bladder, furosemide was injected intravenously 30 minutes prior to the 68Ga-PSMA-11 injection. PET acquisition for the whole-body protocol was in 3D time of flight (TOF) mode, two bed positions with 4 min acquisition time per bed position pelvis to the renal vessels. An additional pelvic frame over 15 minutes was acquired. Based on previous dose reduction calculation we concluded that the 15 min scan would allow us to lower the injected dose to 85 MBq to minimize dose for patients without confirmed cancer (Quantitative performance and optimal regularization parameter in block sequential regularized expectation maximization reconstructions in clinical 68Ga-PSMA PET/MR. Ter Voert EEGW, Muehlematter UJ, Delso G, Pizzuto DA, Müller J, Nagel HW, Burger IA. EJNMMI Res. 2018 Jul 27;8(1):70. doi: 10.1186/s13550-018-0414-4.). Axial FOV was of 25 cm, overlap of 24%, matrix 256x256, 2 iterations, 28 subsets, with sharpIR algorithm (GE Healthcare), and 5 mm filter cutoff. To rule out lymph nodes or distant metastasis, one more frame with 4 minutes frame-time was be performed up to the renal vessels. The MRI protocol included specific sequences covering the pelvis: a high-resolution T1-weighted LAVA-FLEX sequence, T2-weighted fast recovery fast spin-echo sequence in three planes and diffusion weighted images, detailed in the table below.

	Axial DWI EPI (Focus) (Pelvis)	Axial LAVA-FLEX WB (DIXON)	Axial T1w Whole ARC (Pelvis)	Axial T2w FRFSE-XL (Pelvis)	Coronal T2w WB FRFSE-XL	Coronal T2w FRFSE-XL (Pelvis)	Axial DCE (Lava Dyn) (Pelvis)	Ax syn. DWI Focus	Sag T2w FRFSE
Repetition time, TR (ms)	4000	5·6	550	5034	5538	5034	6·361	3500	4678
Echo time, TE (ms)	67·3	1·3-2·7	8·26	120	120	120	2·376	Minimum	120
Flip angle, FA (degrees)	90	12	111	140	111	140	30	-	140
Acquisition matrix	160 x 80	344 x 256	384x384	300x280	288 x 224	300x280	160 x 80	140x70	300x272
Image size (voxels)	256 x 256	512 x 512	512 x 512	512 x 512	512 x 512	512 x 512	288 x 192	-	
Slice thickness (mm)	4	3	5	3·5	5	3·5	4	4	3·5
Signal averages	8	0·68	0·5	2	0·5	4	0·35	-	
b-values (s/mm2) and signal averages	0 (6 av.)<br>400 (8 av.)<br>700(16 av.)							0<br>400<br>1000<br>1500<br>2000	
Diffusion direction	‘All’							All	
Bandwidth (Hz/pixel)	1953	166	62·5	50	90·9	50	62·5	250	50
Acquisition time (mm:ss)	5:41	0:18	1:44	3:27	0:50	3:27	3:27	4:05	3:12

Imaging analysis

The acquired ^{68}Ga-PSMA-11 PET and MRI images were transmitted to a dedicated review workstation (Advantage Workstation, Version 4.6 or 4.7, GE Healthcare), which enables the review of the ^{68}Ga-PSMA-11 PET and the MRI images side by side and in fused mode. All scans were analysed by a double-boarded nuclear medicine physician and radiologist, specialist in pelvic imaging, with 10 and 5 years of experience (IAB, MM), incorporating both the MRI and ^{68}Ga-PSMA-11 PET information. The readers had access to clinical information for the readouts. Dual board certified nuclear physicians and radiologists interpreted the scans, taking zonal anatomy based on T2-MRI into account. Lesions were considered suspicious if there was focal PSMA-uptake higher than local background. Readers were aware of potential pitfalls, such as increased uptake in prostatitis or in the central zone to avoid false-positive findings (Pizzuto et al. The central zone has increased 68Ga-PSMA-11 uptake: «Mickey Mouse ears» can be hot on 68Ga-PSMA-11 PET. EJNMMI, 2018). PSMA-negative lesions seen only on the MRI component were considered as negative.

Table S1 Classification of patients that had up to three lesions with different classifications

	Lesion 1	Lesion 2	Lesion 3	Patient classification
Patients with 2 lesions	TP	FP or FN	---	TP
	FN	FP	---	FN
Patients with 3 lesions	TP	FP or FN	FP or FN	TP
	FN	FP or FN	FP or FN	FN

TP= true positive; FP= false positive; FN= false negative. Patients classified as false positive had only false-positive lesions.

Table S2 Distribution of patients with sigPCa, insigPCa and no cancer, based on biopsy, according to PIRADS, ISUP grade groups and PSMA-PET/MRI result using the criteria for clinically significant prostate cancer as tumours with ISUP grade ≥ 2.

	sigPCa	insigPCa	No cancer
PIRADS			
3	3 (10%)	1 (25%)	3 (43%)
4	17 (55%)	3 (75%)	4 (57%)
5	11 (35%)	0	0
ISUP			
1	0	4 (100%)	-
2	12 (39%)	-	-
3	9 (29%)	-	-
4	8 (26%)	-	-
5	2 (6%)	-	-
PSMA-PET/MRI			
Positive	27 (87%)	1 (25%)	0
Negative	4 (13%)	3 (75%)	7 (100%)
Total	31	4	7

sigPCa = clinically significant prostate cancer; insigPCa = clinically insignificant prostate cancer

Table S3 Performance of PSMA-PET/MRI for biopsy guidance, given patient-based for PSMA-PET/MRI imaging findings and PET-targeted cores, and lesion-based, using the criteria for clinically significant prostate cancer as tumours with ISUP grade ≥ 2.

	Patient-based	Patient-based targeted cores	Lesion-based
Sensitivity	87% (27/31)	68% (21/31)	66% (33/50)
Specificity	91% (10/11)	91% (10/11)	-
PPV	96% (27/28)	75% (21/28)	87% (33/38)
NPV	71% (10/14)	71% (10/14)	-
Accuracy	88% (37/42)	74% (31/42)	-

PPV= positive predictive value; NPV= negative predictive value. For the targeted cores analysis, values were calculated as if patients with a negative PSMA-PET/MRI were not submitted to biopsy and patients with a positive PSMA-PET/MRI underwent

only PSMA-PET/MRI targeted biopsy. Lesion-based specificity and NPV cannot not be calculated since patients with negative PSMA-PET/MRI and no significant cancer on biopsy have, per definition, no lesion.
