# Supplementary material for: Diagnostic performance of 68Ga-PSMA-11 PET/MRI-guided biopsy in patients with suspected prostate cancer: a prospective single-center study
Source: Eur J Nucl Med Mol Imaging. 2021 Feb 23;48(10):3315–24. doi: 10.1007/s00259-021-05261-y (PMC8426229; doi:10.1007/s00259-021-05261-y)
Supplement: Supplementary file 2 — (PDF 275 kb) [file 259_2021_5261_MOESM2_ESM.pdf]

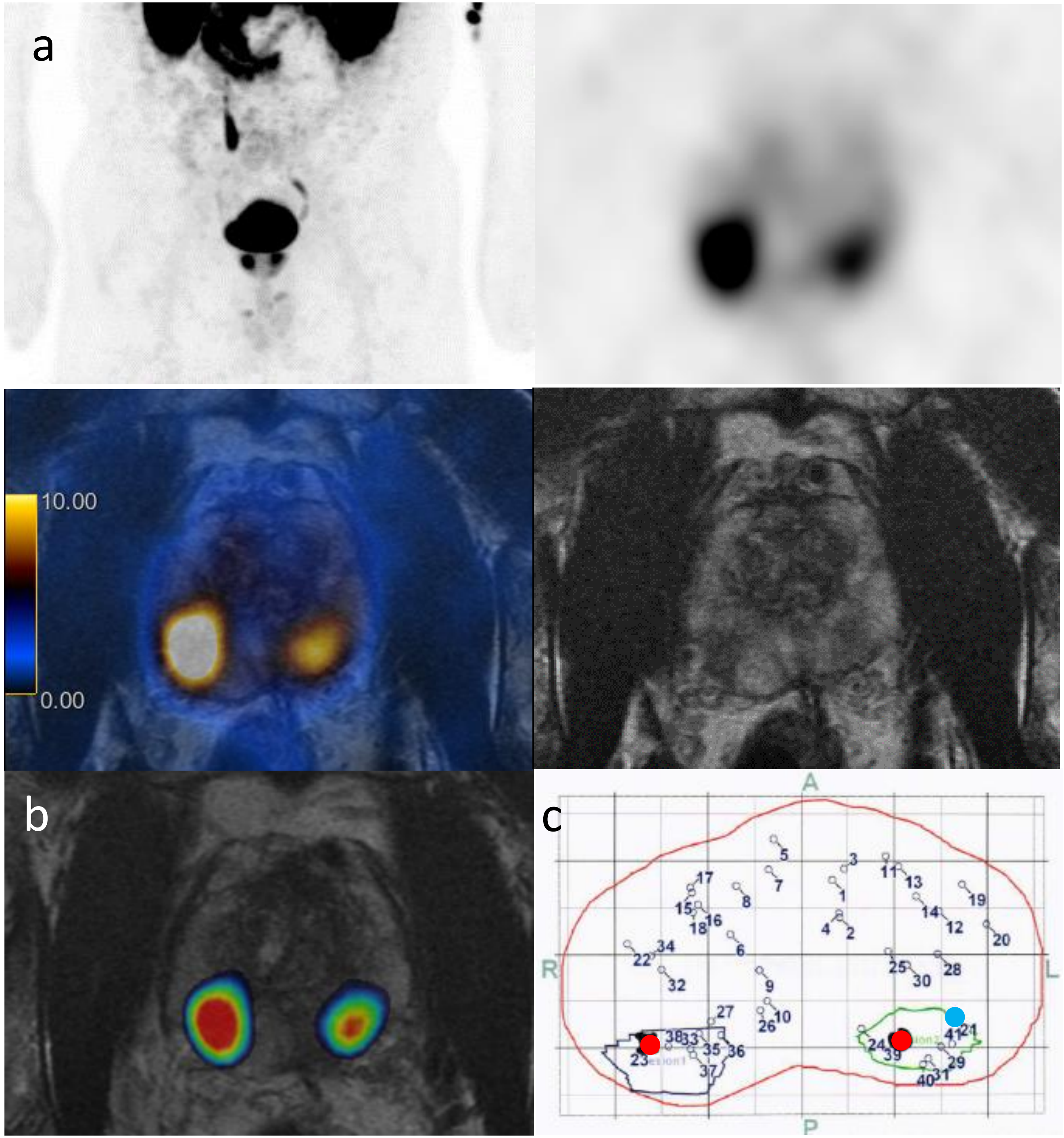

Example of PSMA-PET/MRI targeted biopsy. (A) PSMA-PET/MRI of a 57 years-old patient with prostate specific antigen (PSA) level of 15 ng/ml and a PIRADS 5 lesion on previous multiparametric resonance magnetic imaging (MRI) showing two suspicious lesions with high PSMA uptake (SUV_{max} 19.3 on the right and 13.5 on the left lesion) with hipointense signal on T2-weighted MRI sequence. (B) DICOM file inserted by the urologist in the MRI-US machine, prepared by the nuclear physician who outlined the two suspicious lesions on PET for biopsy targeting. (C) Representative prostate map with targeted lesions delineated in blue and green according to the DICOM file. Each number represent a biopsy sample, the position of needles with clinically significant cancer (ISUP grade 4 on the right and 3 on the left) and clinically insignificant cancer are marked by red and blue dots, respectively.

Diagnostic performance of ^{68}Ga-PSMA-11 PET/MRI-guided biopsy in patients with suspected prostate cancer: a prospective single-center study

Journal: EJNMMI

Authors: *Daniela A. Ferraro, Anton S. Becker, Benedikt Kranzbühler, Iliana Mebert, et al.*

Corresponding author: Irene A. Burger. Department of Nuclear Medicine, University Hospital Zurich, University of Zurich, Zurich, Switzerland and Department of Nuclear Medicine, Kantonsspital Baden, Baden, Switzerland. E-mail: irene.burger@usz.ch
