# Supplementary material for: Diagnostic performance of 68Ga-PSMA-11 PET/MRI-guided biopsy in patients with suspected prostate cancer: a prospective single-center study
Source: Eur J Nucl Med Mol Imaging. 2021 Feb 23;48(10):3315–24. doi: 10.1007/s00259-021-05261-y (PMC8426229; doi:10.1007/s00259-021-05261-y)
Supplement: Supplementary file 4 — (PDF 235 kb) [file 259_2021_5261_MOESM4_ESM.pdf]

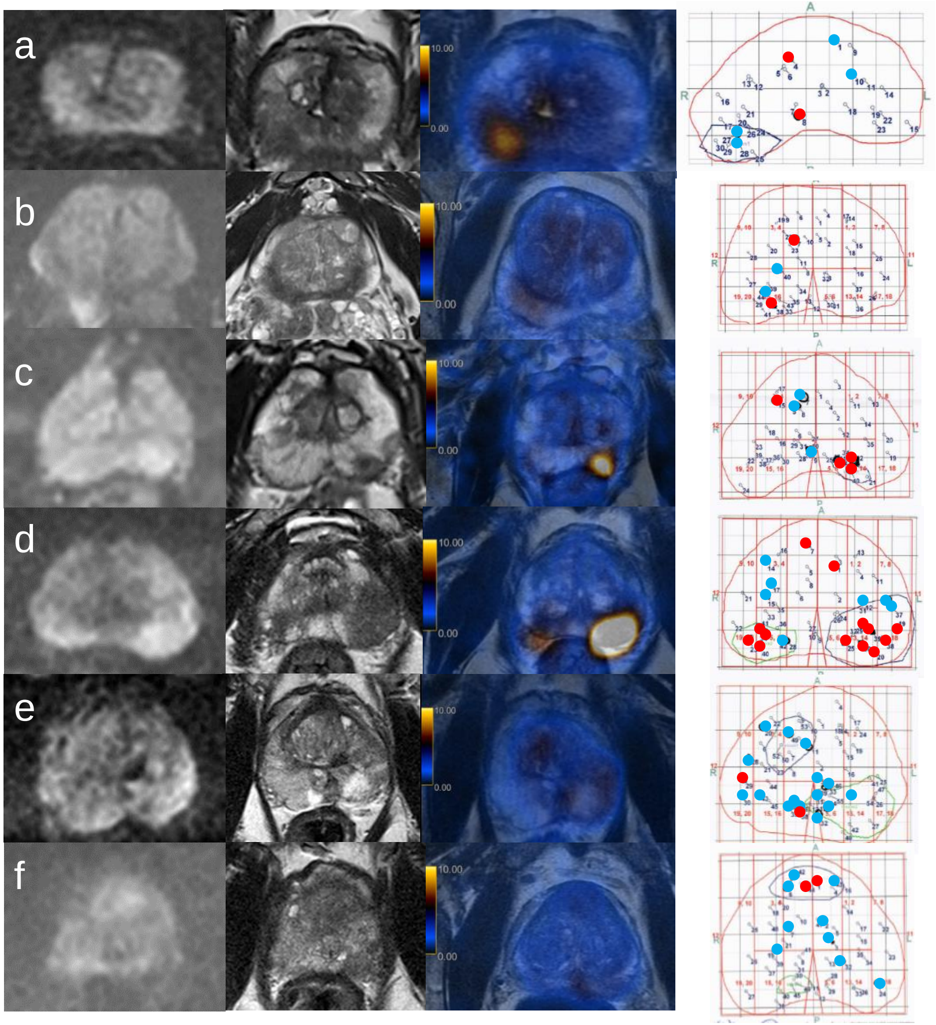


**False-negative PSMA-PET/MRI lesions based on biopsy.** From left to right, prostate magnetic resonance imaging (MRI) sequences diffusion-weighted T2-weighted images, fused PET/MRI and representative pathology map with biopsy results in which red and blue dots correspond to the location of needles with clinically significant cancer and clinically insignificant cancer, respectively. Table 3 shows the biopsy and radical prostatectomy findings for each lesion.

Diagnostic performance of $^{68}$Ga-PSMA-11 PET/MRI-guided biopsy in patients with suspected prostate cancer: a prospective single-center study

Journal: EJNMMI

Authors: *Daniela A. Ferraro, Anton S. Becker, Benedikt Kranzbühler, Iliana Mebert, et al.*

Corresponding author: Irene A. Burger. Department of Nuclear Medicine, University Hospital Zurich, University of Zurich, Zurich, Switzerland and Department of Nuclear Medicine, Kantonsspital Baden, Baden, Switzerland.
E-mail: irene.burger@usz.ch
